# Separating art from the artist: The effect of negative affective knowledge on ERPs and aesthetic experience

**DOI:** 10.1371/journal.pone.0281082

**Published:** 2023-01-31

**Authors:** Hannah Kaube, Anna Eiserbeck, Rasha Abdel Rahman

**Affiliations:** Department of Psychology, Humboldt-Universität zu Berlin, Berlin, Germany; Universitat Autònoma de Barcelona: Universitat Autonoma de Barcelona, SPAIN

## Abstract

Some artists do terrible things. But does knowing something bad about an artist affect the way we perceive the work? Despite increased public interest, this question has yet to be addressed empirically. In this pre-registered study, we used aesthetic ratings and electrophysiological brain responses to shed light on the issue. We found that paintings of artists associated with negative-social biographical knowledge were liked less and found more arousing than paintings of artists associated with neutral information. Such paintings also elicited an enhanced brain response associated with fast and reflexive processing of emotional stimuli (early posterior negativity; EPN). Evaluations of quality and later, more controlled brain responses (late positive potential; LPP) were not affected. Reflecting the complexity of aesthetic experience, this pattern of results became more differentiated when the visual relatedness between the contents of the painting and the artist-related information was taken into account. Overall, our findings suggest that emotional aspects involved in art reception are not spontaneously separated from the artist, whilst evaluative judgments and more elaborate processing may be.

## Introduction

Do we separate art from the artist? Following recent revelations and abuse allegations across multiple artistic disciplines, this age-old question has newly sparked public debate [1–3]. Yet, while our aesthetic experience seems to be contingent on many factors beyond a work’s inherent perceptual properties (e.g. [4–9]), little is known about how art reception is shaped by our affective knowledge about the artist [10]. The present study was designed to contrast the idea that art can be dissociated from moral concerns [11], with the notion that an artwork is treated as if it were an extension of the artist [12]. In a controlled, fully counterbalanced experimental design we investigated whether negative, socially relevant biographical information, which is, prima facie, unrelated to an artist’s working life, influences different outcomes related to aesthetic experience and the neurocognitive processes which underlie them.

Theoretical accounts of art perception suggest that declarative knowledge can play an important, interactive role in shaping the emotional and judgment-based outcomes which form part of an aesthetic experience [5, 7, 13]. Concerning artist-related information or assumptions, behavioural findings have linked artists’ perceived eccentricity to liking ratings and assessments of quality (e.g. [14]). The attitude towards an artwork has also been shown to vary as a function of an artists’ perceived passion and commitment to their work [15]. Building on this, EEG evidence indicates that information about object authenticity, which directly pertains to an artist’s intentions [16], is integrated very rapidly; differential effects can be traced as early as 200ms following stimulus onset [17]. Labelling an artwork as inauthentic also elicits greater activation in the right precuneus and frontopolar cortex [18]. These brain regions are associated with executive functions such as episodic memory retrieval, the integration of different outcomes and relational reasoning [19, 20], thus emphasising the interplay between an artwork’s visual input and higher-order cognitive processes. Taken together, these findings suggest that the neural processing and experience of an artwork is partly rooted in information about its creator.

However, whilst there is tentative evidence to indicate that information referencing the “moral mind” of an artist can affect measures connected to such outcomes [10] direct and conclusive, empirical evidence on this question is lacking. It has been suggested that when viewing art, experiencing negative emotions can increase the intensity of involvement and result in increased feelings of interest and arousal [21]. In line with this, providing non-art experts with sensationalizing information (e.g., *“August Macke’s life was cut short after being called to join the army in WW1”)* increased their electrodermal activity, a measure sensitive to arousal states, while viewing the artwork [22]. More generally, a change in the liking of a stimulus (e.g., a person or an object), after it becomes associated with another strongly valenced stimulus, describes a well-documented affect transfer attributable to evaluative learning (e.g., [23]). In this way, a painting associated with positive expert reviews (e.g., “good”) is evaluated more positively than one which received negative reviews (e.g., “boring”; [24]). Evaluative learning could thus be viewed as a general framework for investigations related to the acquisition of affective knowledge and its effects on object processing. However, beyond the general effects of affect-based learning on valence, further processes related to perception, and other dimensions of stimulus evaluation may also be differentially affected.

For instance, studies on the impact of affective person-related information on face processing indicate that acquiring knowledge about a person’s transgressions or misdeeds not only changes their likeability, but also the way we perceive their face and experience their emotional facial expression (e.g. [25–28]). Moreover, there is evidence to suggest that learning about the affective value of people encompasses both affect-based mechanisms as well as person-attribution processes [29]. Such findings leave room for the possibility that the aesthetic experience, perception and evaluation of an artwork may be impacted by affective, artist-related information in nuanced and diverse ways. The elusive nature of an aesthetic experience and the complexity of the processes underlying it (e.g. [5, 7, 30]), further highlight the need to consider dimensions which are not directly accessible via valence measures [31]. The aim and focus of the present study is therefore to shed light on the interplay between artist-related knowledge and different facets of art reception; by taking the aesthetic, affect-based and person-related mechanisms potentially involved in the acquisition of such knowledge into account.

**Fig 1:**
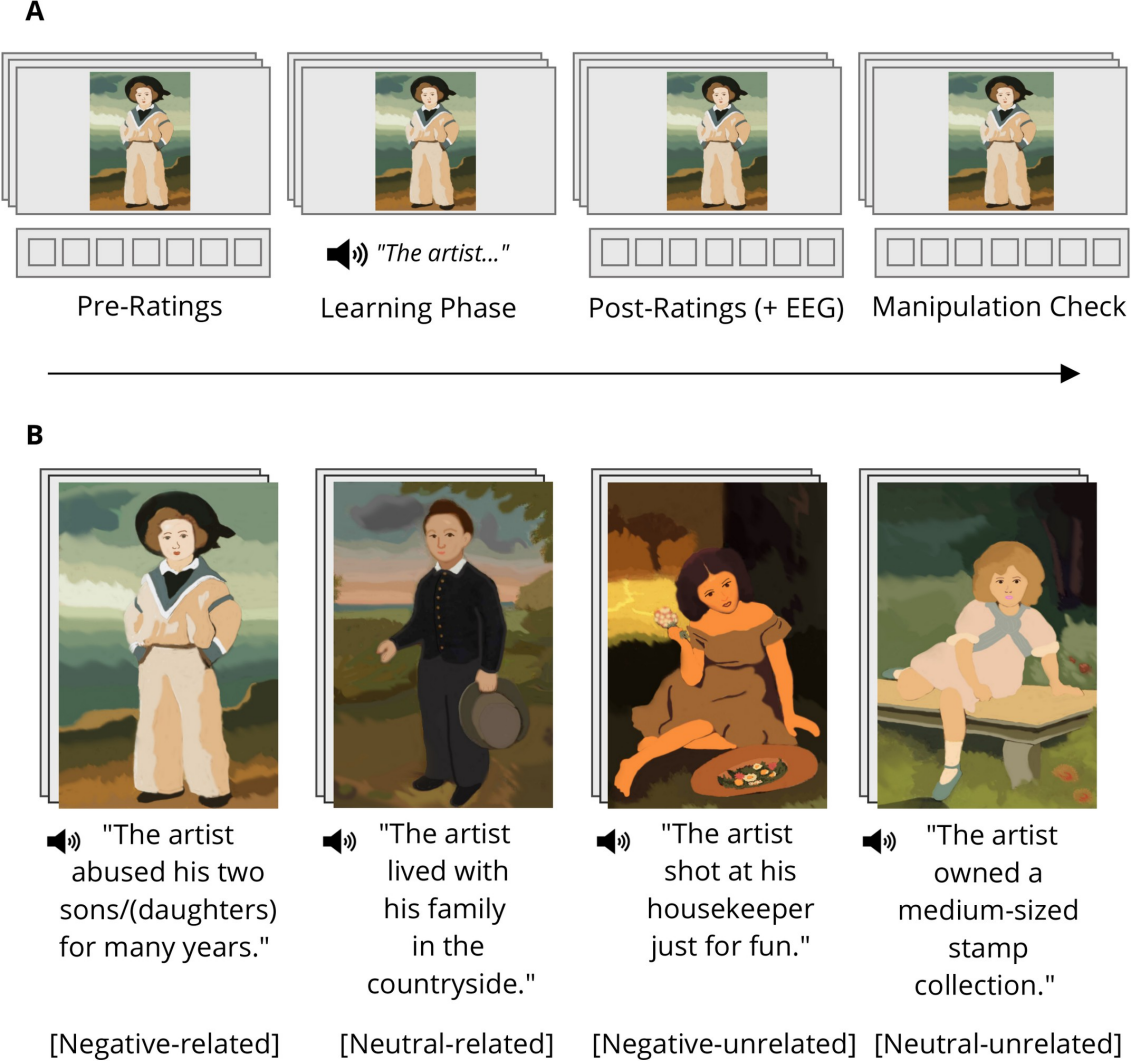
Experimental paradigm. (A) Procedure. All paintings were rated for liking, arousal and quality, before and after knowledge acquisition. The EEG was recorded during the second liking rating. The post-experiment questionnaire measured familiarity with the stimuli, believability of information and ability to recall the information. (B) Schematic illustration of the experimental manipulation. Participants learnt either neutral or negative information about the artist of each painting which was either related or unrelated to the content of the image. The assignment of paintings to the conditions was counterbalanced across participants. To facilitate a within-person comparison of negative and neutral conditions, paintings were matched for content, style and complexity (e.g. paintings of the boys). Matched pairs were coupled with similar, but easily distinguishable pairs (e.g. paintings of the girls). In the experiment, original artworks from different artists were used. Images have been reproduced by the authors for publication purposes. Sentences have been translated from German.

To this end, the present study employed a variant of a well-established social learning paradigm [25, 27, 32, 33], where participants were repeatedly presented with paintings and acquired negative social-affective or neutral biographical information about their artists (see Fig 1). Based on the above outlined theoretical accounts and empirical evidence on art reception, our measures were chosen to cover different components of this process. Specifically, we measured perceptual, emotional and evaluation-based aspects. All paintings were rated by the participants on the dimensions of liking, arousal and perceived quality before and after social-affective or neutral biographical information about the artist was learnt. We expected that paintings of artists associated with negative information would be liked less, induce greater feelings of arousal, and would be judged lower in terms of artistic quality [10, 21, 22].

We recorded electrophysiological brain responses from the EEG during liking ratings post knowledge acquisition, allowing us to unravel the cognitive dynamics of potential knowledge effects as they unfold, with high temporal precision. Drawing on the methodology and results of previous research on face processing, our analyses focused on two electrophysiological markers extracted from the EEG (e.g., [25–28]). The early posterior negativity (EPN) is an index of a fast, reflexive, emotional response to the perception of visual emotional stimuli, such as emotional objects or scenes (e.g., [34–36]). In contrast, the late positive potential (LPP) is associated with slower, more elaborate and controlled processing of motivationally relevant stimuli [37–40]. Both components have been shown to be sensitive to changes in face processing induced by negative biographical knowledge (e.g., [25, 27, 28, 41, 42]). We therefore hypothesized that when viewing paintings, negative affective, compared to neutral knowledge about artists, would lead to more pronounced brain responses related to fast, reflexive (EPN) and later, more elaborate stimulus evaluation (LPP).

An artist’s experiences and beliefs, regardless of their valence, are often depicted within the content of the artwork; thus, the physical image may relate directly to aspects of its creator’s biography. However, not every work in an artists’ oeuvre will evince this type of experiential transfer. An artist with a history of domestic violence, racist convictions or paedophilic crimes can produce a still-life of a bowl of fruit, i.e., an image with an entirely unconnected subject matter. As the visible salience and discernibility of information may affect how individuals view and respond to the work [9, 10], we included relatedness of the visual content to the biographical information as an additional factor. We therefore varied the information provided by valence (negative-social vs neutral) and by whether or not it is relevant to the content of a given painting (related vs unrelated; see Fig 1). To disentangle visual properties of the paintings from knowledge effects of interest, the assignment of paintings to experimental conditions was counterbalanced across participants. We expected information which is visually discernible (i.e., related) to be more relevant to the interpretation of the visual input and therefore lead to more pronounced effects in measures associated with the emotional processing of the images: liking, arousal, EPN and LPP amplitude. However, we did not expect a modulating effect of relatedness on quality judgments, as assessments of this dimension should be less dependent on the specific content of the image.

## Method

### Design overview

The study was based on a 2 by 2 design with valence of information (neutral vs. social-negative) and content relatedness (related vs. unrelated) as within-subject factors; yielding a total of 4 conditions: neutral–unrelated; negative–unrelated; neutral–related, negative–related. The assignment of images to conditions was fully counterbalanced across participants. Our dependent variables consisted of behavioural (liking, arousal and quality) ratings and ERP components (specifically, EPN and LPP).

### Sample size

To assess the required sample size, a behavioural pilot study (N = 8; mean age = 26; range of 18–32; 5 women) was conducted. The fixed effect of negative, compared to neutral knowledge on liking ratings was estimated. Liking was predicted by knowledge, relatedness and the interaction between both factors (as fixed effects) and random intercepts were modelled for participants and items. The SIMR package in R [43] was used to run an *a priori* power analysis based on the resulting effect size (*b* = -0.40). Simulations were run 1000 times on different potential sample sizes, all of which were multiples of 8, to accommodate the perimeters of our balanced design. Results indicated that a sample of 24 would be necessary to detect a knowledge effect on liking ratings with an expected power of 93.3%, 95% CI [91.57, 94.77]. As ERP effects were expected to be smaller than behavioural effects, we included 32 participants in our study. This sample size has also previously been sufficient to detect both main knowledge effects and interaction effects on EPN and LPP amplitudes, by studies employing a similar experimental paradigm [25, 27, 32, 33].

### Participants

As previous literature indicates that experts view and evaluate art differently to non-experts (e.g., [4, 22, 44]), individuals with a formal background in the visual arts (i.e., individuals who indicated that they had studied, or were currently studying art or art history) were not recruited. One participant was replaced due to excessive movement artefacts and three due to poor quality EEG data. No participants were excluded based on learning inaccuracy or familiarity with the stimulus material (see Manipulation Check). The final sample consisted of 32 (mean age = 26.13; range of 18–37; 18 women) native German speakers with normal or corrected-to-normal eyesight and normal colour vision. Participants received monetary compensation (10€ per hour) or credit points. Before participating, all individuals provided informed consent. The study was approved by the Ethics Committee of Humboldt University’s psychological institute and complied with the standards set by the Declaration of Helsinki.

### Materials

For the visual stimuli, 32 colour images of paintings, spanning a range of different artistic styles and eras, were selected. The selection only contained paintings which art novices are unlikely to be familiar with. Purely abstract works with no discernible forms were excluded, as constructing stories with related and unrelated content was rendered too subjective. The selection consisted of 16 pairs of paintings, matched (subjectively) in terms of style, content and complexity. The matching procedure required all authors to be in agreement regarding the resultant pairs. Matched paintings were then coupled with other similar, but easily distinguishable pairs, resulting in eight sets of four pictures (see Fig 1 for an example set of pictures).

The paintings were resized and processed to ensure identical dimensions within the sets. Paintings from different sets varied in their dimensionality to preserve the aspect ratios of the original images. Across the sets, all pictures were normed to have the same area and resolution when presented: 56,400 pixels with 72 dpi. To avoid saturation of negatively valenced stimuli and increase believability of the fictitious stories associated with the paintings, eight further pictures were selected as filler items associated with positive information. These were not included in further analyses. Visual angles ranged from 4.64° to 6.60° horizontally and from 4.96° to 7.07° vertically depending on the set. Viewing distance was set at 70 cm.

For each set of paintings, a corresponding set of four sentences, one for each of the experimental conditions was constructed (see Fig 1). The relatedness between sentences and images was assessed independently by three raters with no involvement in the study. The intra-class correlation coefficient, computed using the two-way random effect models and “single rater” unit, showed a good level of absolute agreement between ratings [45], kappa = 0.81, 95% CI [0.74, 0.86]. Positive affective information was also created for the filler pictures. The biographical information was recorded and presented to the subjects audibly. During the recording, the (male) speaker was instructed to keep his tone neutral and unemotional for all sentences. None of the stories alluded to style, ability, prestige or artistic competence. All sentences had the same grammatical structure and there was a maximal difference of one word in length between sentences within a set.

A pilot rating study was conducted (N = 26; mean age = 27; range of 21–39; 20 women), to ensure that the related and unrelated conditions did not differ in terms of arousal and valence. Stories were rated on a 7-point scale from “very negative” to “very positive” and from “not at all arousing” to “very arousing”. An alpha level of .05 was used to compute a repeated-measures analysis of variance (ANOVA). The valence ratings varied only as a function of the valence of the information *F*(1, 25) = 956.29, *p* < .001 (negative: *M* = 1.52, *SD* = 0.38; neutral: *M* = 4.11, *SD* = 0.22), but there was no difference between related (*M* = 2.83, *SD* = 1.34) and unrelated sentences (*M* = 2.80, *SD* = 1.35), *F*(1,25) = 1.35, *p* = .256, nor did the two conditions interact *F*(1, 25) = 0.14, *p* = .713. Paralleling this, whilst arousal ratings differed significantly between the neutral (*M* = 1.52, *SD* = 0.77) and negative (*M* = 5.19, *SD* = 1.00) sentences *F*(1, 25) = 340.32, *p* < .001, no main effect of relatedness was found, *F*(1, 25) = 3.38, *p* = .078 (related: *M* = 3.30, *SD* = 2.00; unrelated: *M* = 3.41, *SD* = 2.10) and the interaction between the two conditions remained non-significant *F*(1, 25) = 1.90, *p* = .180. This precludes the possibility that any observable effects found, were due to affective differences between the related and unrelated conditions.

### Procedure

The experiment took three and a half hours in total, including the preparation and application of the EEG. Participants were tested individually and were seated in a shielded room to reduce electromagnetic interference for the main part of the experiment. The follow-up questionnaire was conducted online at a different computer located in the adjoining room. At the beginning of the experiment, participants were required to rate each picture. A fixation cross was displayed in the centre of a grey screen for 0.5s, followed by one of the paintings, presented for 1.5s. The paintings were then replaced by the scale for one of the three rating tasks. These tasks were conducted block-wise. Within each rating block, paintings were presented in a randomised order. First, participants were asked to rate the paintings for the dimension of liking on a 7-point scale, ranging from “not at all” to “very much.” Participants then rated the pictures for the dimensions of arousal (“not at all moving” to “very moving”) and artistic quality (“very bad” to “very good”) on a 7-point scale, order counterbalanced across participants. For all three tasks, participants were instructed to answer quickly and intuitively, but the responses were not subjected to a time limit.

During the learning phase, each painting was presented on the screen while participants listened to the pre-recorded information detailing an aspect of the artist’s alleged biography. The allocation of paintings to the experimental conditions (related vs. unrelated and neutral vs. negative) were fully counterbalanced across participants. The positive fillers maintained the same painting and information combination for all participants. To ease the acquisition of knowledge, the presented paintings-information pairings were initially split into mini blocks of equal size (5 pairings per block). The allocation of paintings to a block and the order of presentation was randomised for each participant. The size of the blocks was then increased, until participants were presented with the entire collection of picture-information pairings consecutively. All paintings and respective stories were presented a total of seven times. Dispersed throughout the learning phase, participants were asked control questions to ensure that the stimuli were attended to carefully, as per instruction (e.g., “is the artist’s behaviour something most people would consider ordinary?”). During the test phase, the pictures were presented without the information and the EEG was recorded. The trials were identical to those participants encountered during the liking ratings at the start of the experiment. Each picture was presented a total of eight times in randomised order, resulting in 64 trials per condition. At the end of the test phase, the artworks were again rated for arousal and perceptions of quality in counterbalanced order.

Upon completing the experiment, participants were shown each experimentally relevant image again in an online questionnaire. Familiarity with the images was assessed via a 7-point scale (1- definitely not seen painting before today, 7- definitely seen painting before today). Participants were also required to recall the affective nature of the information associated with each image (classification performance below 50% was predefined as an exclusion criterion). To implicitly determine story believability, participants were asked to indicate whether the learnt information was novel. The options "I think I have heard this information before" and "I do not think I have heard this information before" were coded as "believed". The option "I do not believe the information" was coded as "not believed." Participants were also asked whether they knew the name of the artist of the image (options: "no" and an open-ended text field). At the end of the questionnaire, participants’ interest in art was measured on a 7-point scale (“how interested are you in art?”; 1-not at all to 7-very).

### EEG recording and data analysis

The EEG was recorded using Ag/AgCl electrodes from 62 scalp sites according to the extended 10/20 system, referenced to the left mastoid. The sampling rate was 500 Hz. Electrode impedance was kept below 10 kOhm. An external electrode attached below the left eye measured the electrooculogram generated from eye movements and blinks. In a short calibration procedure, prototypical eye movements were obtained to correct for ocular artifacts. Offline, the continuous EEG was re-referenced to a common average reference and filtered (no low cut-off, high cut-off 40Hz). Ocular artifacts were removed by estimating spatiotemporal dipole distributions in BESA [46]. Further artifacts (defined as segments containing amplitude values ± 150 μV or gradients > 50 μV) were also excluded from further analyses. The corrected EEG data was segmented into epochs, starting 100ms prior to picture onset and continuing for 1000ms during presentation of the picture. The pre-stimulus baseline was defined as 100ms prior to picture onset. No electrodes were interpolated and on average 1% of all trials were rejected across participants.

To analyse the data, a processing pipeline focusing on single-trial based analyses using linear mixed models (LMM) was implemented [47]. ERPs were averaged across the time windows of interest at topographical sites typically associated with the components. The EPN was analysed in a time frame of 250-350ms (electrode sites: PO7, PO8, PO9, PO10, TP9, TP10; [25]) and the later elicited LPP between 400 and 700ms (electrode sites: Pz, Cz, C1, C2, CP1, CP2; [25]). The single-trial based LMM analyses were performed using the lme4 package version 1.1–20 [48] in R and p-values were calculated via the lmerTest package version 3.0–1 [49]. An alpha level of .05 was used for all statistical tests.

For the LMM analyses of ratings and ERP components of interest, each dependent variable (liking, arousal, quality, EPN, LPP) was modelled separately, using valence of knowledge (social- negative vs neutral), relatedness to the picture (related vs unrelated) and the interaction between the two factors as fixed effects. Random intercepts were modelled for participants and items. To account for the fact that experimental effects can vary heterogeneously across different items and participants [47], random slopes were modelled for the predictors. Hereby, we aimed to take the maximum plausible random effects structure into account [50]. Random slopes which were not supported by the data and prevented model convergence were assessed via singular value decomposition and removed. Although the liking ratings were repeated several times during the test phase to accommodate ERP methodology, only the first trial was included in the liking analyses. Post-hoc models including art interest as a centered covariate were also analysed for each dependent measure and can be found in Tables S1 to S5 in S1 File.

### Manipulation check

On average, participants correctly remembered the affective nature of 96% of the information (range: 66–100%) in the post-experiment questionnaire. Only 6% of the information was explicitly disbelieved (range: 0–46.8%). Across the sample, the feeling of familiarity of the pictures was collectively low (*M* = 2.13, *SD* = 1.59). Analysis of variance (ANOVA) with an alpha-level of .05, showed that feeling of familiarity did not vary as a function of valence, *F*(1,31) = 0.21, *p* = .648, or relatedness of information *F*(1,31) = 2.57, *p* = .119. Moreover, with one exception, none of the participants could correctly name any of the artists of the paintings (<1% of all cases). This indicates that our experimental manipulation and stimuli selection was successful.

## Results

### Aesthetic ratings

**Fig 2:**
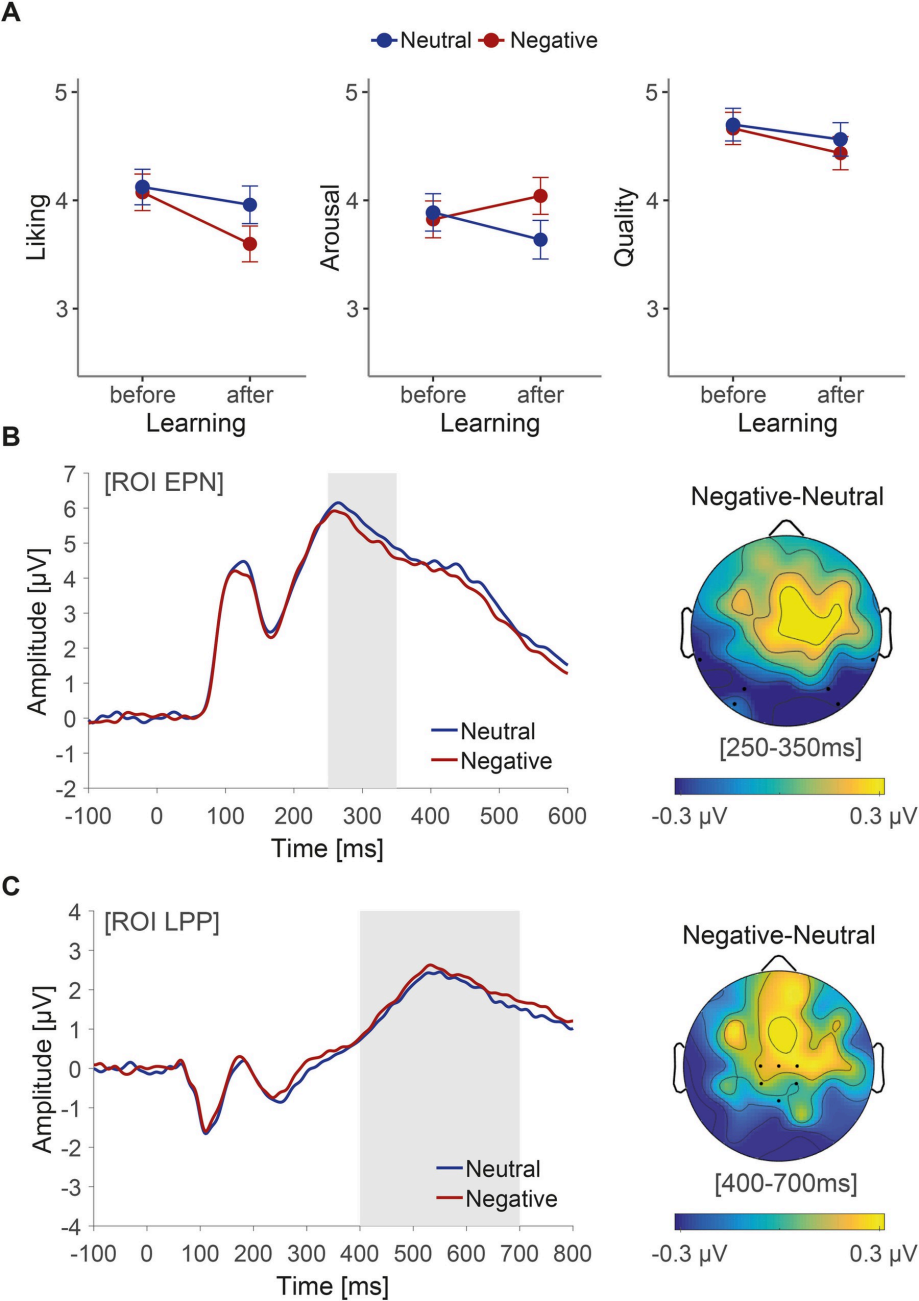
Effect of biographical knowledge on reception of artworks. (A) Ratings of liking, arousal and quality before and after the acquisition of knowledge. Range of scales was 1 to 7. Higher numbers reflect more liking, greater arousal and greater judgments of quality. Error bars represent 95% confidence intervals. (B) Grand average event-related potentials at the occipito-temporal pooled region of interest [ROI] associated with the EPN component. The topographical distribution shows that the difference between negative and neutral knowledge effects resulted in a posterior negativity between 250-350ms (grey area). (C) Grand average event-related potentials at the centro-parietal pooled region of interest [ROI] associated with the LPP component. The difference between negative and neutral knowledge effects did not reach significance between 400-700ms (grey area).

To compare baseline ratings with post knowledge acquisition ratings, we nested the factors knowledge and relatedness within the fixed effect “time of rating” (ratings obtained before vs. after the learning phase). Fig 2A provides a graphical overview of the results. Before learning, paintings were evaluated the same across conditions on all three dimensions (see Tables S6-S8 in S1 File for full statistical output). After learning negative as compared to neutral information about the artist, paintings were liked less (*b* = -0.36, *t* = -3.41, *SE* = 0.11, *p* = .001) and found more arousing (*b* = 0.40, *t* = 3.60, *SE* = 0.11, *p* = .0006), whereas no difference was observed for ratings of quality (*b* = -0.13, *t* = -1.50, *SE* = 0.08, *p* = .134). Relatedness to content and valence of knowledge did not interactively affect any of the aesthetic ratings in these models (liking: *b* = -0.20, *t* = -0.78, *SE* = 0.26, *p* = .440; arousal: *b* = 0.02, *t* = 0.08, *SE* = 0.25, *p* = .939; quality: *b* = 0.01, *t* = 0.07, *SE* = 0.18, *p* = .947).

**Fig 3:**
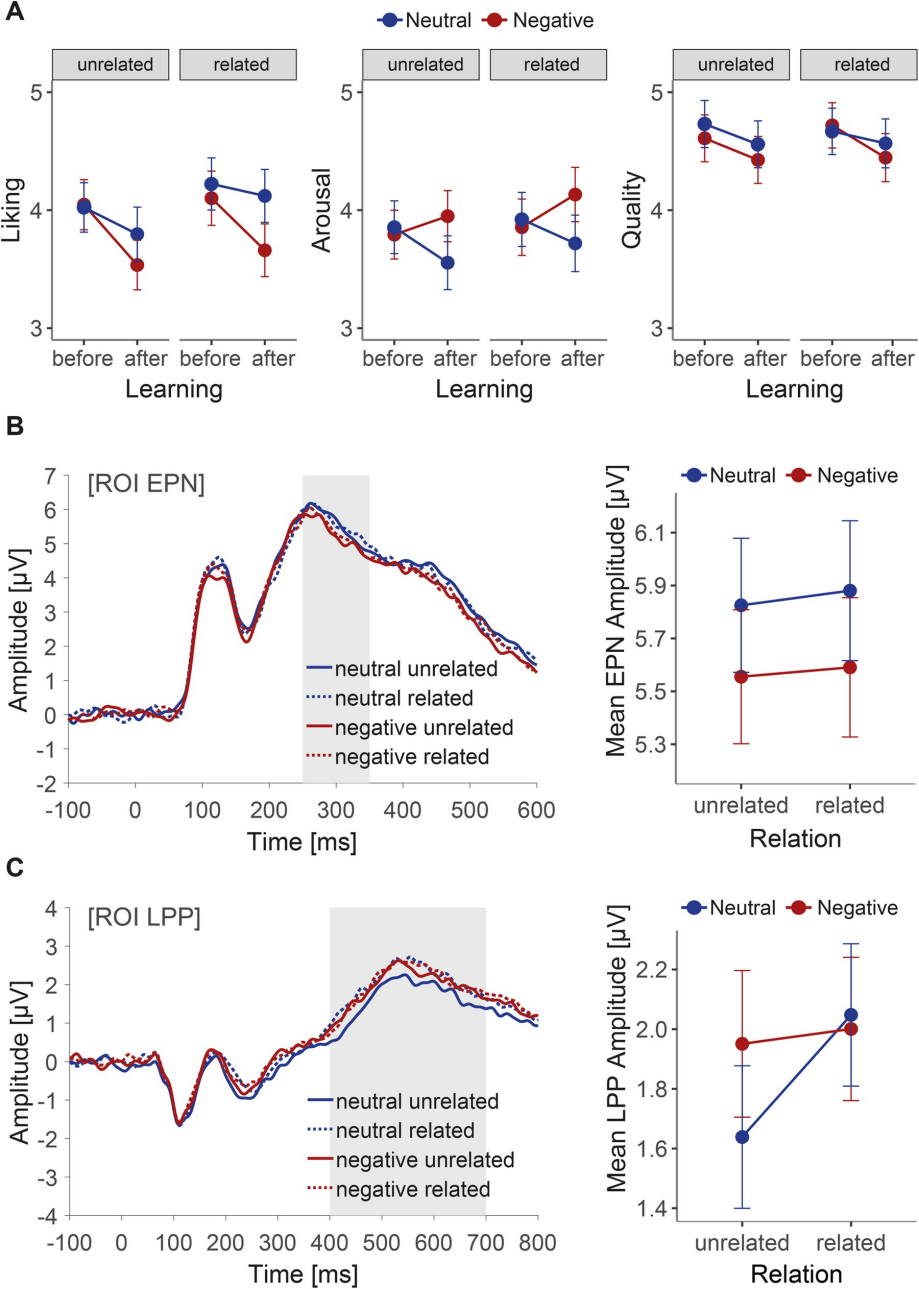
Effects of biographical knowledge and relatedness between knowledge and contents of paintings on reception of artworks. (A) Ratings of liking, arousal and quality before and after the acquisition of knowledge. Range of scales was 1 to 7. Higher numbers reflect more liking, greater arousal and greater judgments of quality. Error bars represent 95% confidence intervals. (B) Grand average event-related potentials at the occipito-temporal pooled region of interest [ROI] associated with the EPN component. Mean amplitude (μV) plot shows no difference between related and unrelated conditions. (C) Grand average event-related potentials at the centro-parietal pooled region of interest [ROI] associated with the LPP component. Mean amplitude (μV) plot shows (non-significant) trend of difference between related and unrelated conditions when the knowledge is neutral.

To provide a clearer understanding of the hypothesised role of relatedness, further models were analysed, nesting knowledge and time of rating within the factor relatedness for each behavioural outcome (see Tables S9-S11 in S1 File). For the liking task, a significant effect of negative (compared to neutral) knowledge was found for information which related to image content (*b = -0*.*46*, *t* = -3.20, *SE* = 0.14, *p* < .01). When the identical information was coupled with unrelated images, only a trend in the same direction was revealed (*b = -0*.*26*, *t* = -1.82, *SE* = 0.14, *p =* .07). Arousal ratings were significantly affected by knowledge in both related (*b = -0*.*41*, *t* = 2.73, *SE* = 0.15, *p <* .01) and unrelated conditions (*b = -0*.*39*, *t* = 2.60, *SE* = 0.15, *p <* .05), whilst ratings of quality remained unaffected by knowledge regardless of relatedness (related: *b = -0*.*12*, *t* = -1.02, *SE* = 0.12, *p* = .306; unrelated: *b = -0*.*13*, *t* = -1.12, *SE* = 0.12, *p* = .262). Fig 3A illustrates the effects of the factors knowledge and relatedness on the behavioural dimensions.

A post-hoc analysis including all liking trial repetitions was calculated to investigate the stability of the found knowledge effect on the dimension of liking (see Table S12 in S1 File). Results demonstrated that the effect of knowledge on liking ratings remained robust when trial repetitions were included as a further factor in the model: *b = -0*.*37*, *t* = -3.51, *SE* = 0.11, *p* < .001. Indeed, trial repetitions did not affect the ratings significantly (*b* = -0.008, *t* = -1.20, *SE* = 0.007, *p* = .231), indicating that liking ratings did not return to the baseline level measured before the acquisition of knowledge.

### Event-related potentials

As shown in Fig 2B, an enhanced EPN was found at posterior electrodes in a time window of 250 to 350ms after stimulus onset for paintings of artists associated with negative, compared to neutral, biographical knowledge (*b* = -0.27, *t* = -2.32, *SE* = 0.11 *p* = .022). The effect of knowledge was not modulated by the factor relatedness (*b* = -0.02, *t* = -0.08, *SE* = 0.29, *p* = .936). At centro-parietal electrodes (Fig 2C), no main effect of knowledge and no interaction between knowledge and relatedness was found between 400 and 700ms (*b* = 0.13, *t* = 1.05, *SE* = 0.13, *p* = .303 and *b* = -0.35, *t* = -1.38, *SE* = 0.25, *p* = .174, respectively). See Tables S13 and S14 in S1 File for full statistical output. Post-hoc analyses were conducted to investigate whether reaction time (i.e., judgment latencies) or trial repetitions confounded these results or affected the predictors. Neither covariate was found to have an interactive effect on EPN or LPP amplitude, suggesting that neither motor confounds, nor habituation effects systematically explain the findings (see Tables S15-S18 in S1 File).

Turning to the secondary hypotheses (see Fig 3B), nesting the factor knowledge within the relatedness condition revealed that the effect of negative (compared to neutral) knowledge on EPN amplitude was similar in size and direction in the related (*b* = -0.28, *t* = -1.76, *SE* = 0.16, *p* = .081) and the unrelated condition (*b* = -0.25, *t* = -1.62, *SE* = 0.16, *p* = .107), although in both cases the effect did not reach statistical significance, due to a reduction in power when analysing the effect of knowledge separately within each condition, i.e. for only half of the trials. Conversely, a differentiated pattern of results was found in the LPP component (Fig 3C). A near significant effect of knowledge could be observed on LPP amplitude in the unrelated condition (*b* = 0.31, *t* = 1.92, *SE* = 0.16, *p* = .058) but not in the related condition (*b* = -0.04, *t* = -0.28, *SE* = 0.16, *p* = .781). See Tables S19 and S20 in S1 File.

## Discussion

Using aesthetic ratings and neurocognitive measures, the present study investigated the influence of affective knowledge about artists on different facets related to the reception of their artworks. Previous literature demonstrates that aesthetic outcomes are dependent on a myriad of intertwined personal and contextual characteristics (e.g., [5, 7]); our experimental design allowed us to empirically narrow the focus to two factors; negative-social biographical knowledge and relatedness of this knowledge to image content. To control for bottom-up visual differences, paintings were matched, and information-painting combinations were presented within a fully counterbalanced learning paradigm. Modelling random effect structures, specifically by-item and by-participant random slopes, further accounted for additional sources of variance [47]. We were therefore able to disentangle the influence of intraindividual and interindividual differences in responses to the paintings from the contextual effects of interest.

To summarize our main findings, paintings by allegedly “bad” artists, were liked less and found more arousing than paintings by artists associated with emotionally neutral information. Underscoring this finding, fast and reflexive brain responses distinguished between the two conditions as early as 250 to 350ms. Thus, social-affective knowledge about the artist is integrated rapidly and influences early facets of art reception. Corresponding to the temporal aspects of this finding, contextual information about authenticity also begins to influence electrocortical activity around 200 to 300ms [17]. Thus, we appear to view an artwork in light of what we know about the artist, indicating that we do not spontaneously separate our perceptual-emotional experience of visual art from affective knowledge about the artist. Extending the view that negative emotions provoked by an artwork can lead to a more intense and moving experience [21], we show that emotional responses can be evoked not only directly by the piece of art, but also indirectly by our knowledge about the artist.

Whilst the behavioural changes in arousal and liking ratings dovetail with findings from evaluative learning literature (e.g., [23, 51, 52]), the lack of change in judgments of quality is harder to delineate within the same framework. Specifically, such investigations generally do not extend to other object appraisals, making it unclear whether a change in liking should also be accompanied by a change in evaluations of other attributes, in this case, judgments of quality [23]. In line with theoretical aesthetic accounts however (e.g., [5]), our behavioural results denote a relative independence between different outcomes underpinning an aesthetic experience. As previously suggested [7], this dissociation may indicate an increase in emotional distance when individuals focus on formal qualities of an artwork. Such accounts are of particular practical merit, as they allow for the possibility of an individual disliking a painting they judge to be of a high qualitative standard, or to be incredibly moved by a single splash of colour on a canvas. In the present study, when judging the quality of the paintings, our participants may have been less emotionally engaged with the artworks, rendering the affective information less salient or informative. Substantiating this perspective, previous empirical research (e.g. [53–55]), has also demonstrated a heterogenous effect of the same contextual information on different aesthetic facets and dimensions.

Our results do not confirm that later brain responses related to elaborate and more controlled processing (LPP) are affected by negative-social biographical knowledge, indicating that relatively slow and more reflective processes during art reception may not be spontaneously influenced by such information. Unlike the reflexive EPN (e.g., [34]), the LPP reflects processes which are sensitive to the personal (e.g., [28, 41]), communicative [56] and task-related [57, 58] relevance of emotional or emotionally imbued stimuli. Mirroring a real-life interaction with art, we did not instruct our participants to consciously focus on the information they had learnt about the artists during their ratings, possibly pre-empting a more sustained and elaborate evaluation at later processing stages. Moreover, a liking judgment, as a task, may not require inferential reflection, as an outcome is spontaneously available (e.g., [59]).

Compounding this, participants in our study had no formal training or background in the visual arts; the inherent personal relevance of the material may therefore have been minimal. As such, a continued, motivated processing of the paintings may not have been requisite to our collective sample. Providing tentative support for this, including the covariate art interest in LMM analyses (see Table S5 in S1 File), revealed that LPP amplitude was significantly more pronounced when participants with high art interest learnt negative, compared to neutral information about the artist of a painting. Individuals who are more interested in art may therefore be more implicitly motivated to sustain the integration of information during later processing stages, as the stimuli are more intrinsically relevant. In line with this, neuroscientific evidence shows that when making an aesthetic judgment, expertise allows individuals to access stored knowledge more readily [60] and LPP amplitude is more pronounced for artists than non-artists [4]. Future research may wish to investigate the role of affective knowledge about artists, whilst emphasising factors such as intrinsic relevance, art interest and expertise.

Mirroring the complexity and multifaceted nature of art reception (e.g., [5, 7, 13, 18]), investigating the role of relatedness revealed an interesting and differentiated pattern of findings. Whilst no significant difference was found between related and unrelated information in the negative condition for quality judgments, liking ratings were significantly affected by negative knowledge which pertained to the subject depicted in the image, but only a trend could be found for negative knowledge in the unrelated condition. As related and unrelated information was matched for both arousal and valence (see Method), the differentiated liking ratings may indicate that the emotional processes underpinning aesthetic outcomes are more intricate than the mechanisms associated with a general evaluative transference of valence from one (dis)liked stimulus to another (for review and meta-analyses see [23, 51]). Evaluative learning therefore seems to constitute an important underlying mechanism at play, but cannot solely account for the complexity of the found results. In this way, our findings corroborate theoretical and empirical literature emphasising the compositeness of an aesthetic experience and delineating its difference to the perception and experience of objects in an everyday context (e.g., [5, 21, 30, 61–64]).

The finding that liking ratings were more affected by negative information which was related, as opposed to unrelated to the content of an image, also coincides with research indicating the dependence of aesthetic outcomes on an individual’s ability to comprehend, interpret, or find meaning in an artwork (e.g., [5, 7, 9, 54, 65, 66]). For example, providing participants with content-relevant information, specifically descriptive titles, elevates their understanding or the perceived meaningfulness of an artwork (e.g., [54, 65, 66]), which in turn influences aesthetic appreciation and hedonic value conferred to painting (e.g., [5, 54, 66]). The ability to discern meaning is therefore an important aspect contributing to the overall aesthetic experience of an artwork. In the present study, the provision of negative information, which was directly observable in a painting, may have furthered participants’ understanding to the detriment of the hedonic value of the artwork. As previously suggested [66], we therefore show that content-relevant information can have a decremental effect on liking, rather than a positive one, if the information facilitates a negative interpretation about the circumstances surrounding its production.

Examining the effect of knowledge on the EPN amplitude separately within the related and unrelated conditions did not indicate differences in the size and direction of effects. Conversely, a near significant effect of knowledge could be observed on LPP amplitude in the unrelated condition but not in the related condition. The unexpected trend appears to be driven by the comparatively low amplitude elicited by images associated with neutral information which bears no relevance to the image’s content, as compared to amplitudes for images associated with negative and/or related information (see Fig 3C). This combination of factors, i.e., information that is both neutral and unrelated, may therefore culminate in stimuli with particularly low motivational significance, a property assumed to be necessary for the sustained engagement of attentional resources and elaborated processing (e.g., [37, 39, 40]). Future research could further investigate the role of motivational significance on the perception of artworks by increasing the relevance of the task during which the EEG is recorded (e.g., [57, 58]), explicitly comparing participants with different levels of expertise (e.g., [4]) or increasing the relevance of the stimuli (e.g., using famous paintings or supplying information about well-known artists which participants are likely to be familiar with).

Outside of the laboratory, biographical information about an artist could be learned from a variety of different sources (e.g., media, gossip, label at an exhibition), with a varying intensity of exposure (e.g., short or long media coverage). In contrast to most experimental aesthetic studies which either once present information simultaneously with, or just before, the visual stimuli (e.g. [8–10, 17]), our intensive learning-paradigm enabled us to ascertain the effect of long(er)-term affective knowledge. This allowed us to approach verbal learning as typified by repeated exposure to media sources and news articles which showcase examples of artwork when presenting biographical information. The methodology used is therefore reflective of a common and relevant, albeit specific variant of real-life social learning. Splitting the learning procedure into two successive phases, such that participants learn to associate the artists with the information and the paintings separately, would provide an alternative experimental framework in which to investigate other ways such knowledge is acquired. Increasing the sample size would also help overcome power-related issues, which may be making the role of relatedness harder to interpret.

Although the present study measured different dimensions involved in the formation of an aesthetic experience, the three behavioural ratings obtained do not cover the full spectrum of aesthetic impressions and emotions evoked by a work of art. A host of literature points to the many varied and nuanced responses potentially elicited by an interaction with an artwork (e.g., disgust, boredom, awe, unease, humour, pleasure, sadness; [67–70]) and future research investigating the effect of negative biographical knowledge on aesthetic outcomes would benefit from employing more differentiated measurement scales (e.g., the Aesthetic Emotions Scale: [71]). Moreover, beyond the influence of contextual information, the actual physical context in which a painting is viewed also affects a person’s experience of it (e.g., [5]). Compared to a gallery setting, factors such as lighting, frames, barriers and displays are necessarily controlled in a laboratory [72]. Differences in hedonic value and viewing behaviour have previously been reported between participants attending to paintings in these contexts [73, 74]. Social interactions and group attendance also appear to play a decisive, though not necessarily positive role on the experience of, and engagement with, artworks [75, 76]. Such additional factors cannot be accounted for by the present study and would also provide fruitful ground for future research.

Returning to the initial question—do we separate art from the artist?- our study reveals evidence that perceptual-emotional aspects of art reception are shaped by our knowledge about the artist. In contrast, judgments of quality and later, more controlled brain responses do not seem to be influenced in the same way. Art is neither conceived, nor received in a vacuum: the interaction between art, artist and viewer needs to be understood within an interdisciplinary framework [77]. The results of our study reflect this necessity and add to the growing body of literature exploring the interface between top-down and bottom-up integration supposedly involved in experiencing art (e.g., [7, 8, 17]). Moreover, in light of recent allegations and revelations, understanding the effect of negative information about artists has become a topic of political and social debate. To date, this discussion has been led in mainly theoretical terms with scant scientific foundation. We demonstrate that neurocognitive and experimental insights can help facilitate a shift to a more empirical frame of reference.

## Supporting information

S1 File Supporting tables with full output. (PDF)
