# Supplementary material for: Separating art from the artist: The effect of negative affective knowledge on ERPs and aesthetic experience
Source: PLoS One. 2023 Jan 31;18(1):e0281082. doi: 10.1371/journal.pone.0281082 (PMC9888721; doi:10.1371/journal.pone.0281082)
Supplement: S1 File — (PDF) [file pone.0281082.s001.pdf]

Supplementary Material

Table S1.

Liking rating: Effects of biographical knowledge, relatedness and covariate art interest before and after knowledge acquisition.

Variable	*b*	*SE*	*t*	*p*
Intercept	3.94	0.14	28.51	<.001***
Learning(Post-Pre)	-0.32	0.07	-4.83	<.001***
Pre/Knowledge(Neg-Neu)	-0.05	0.11	-0.46	0.646
Post/Knowledge(Neg-Neu)	-0.36	0.11	-3.42	<.001***
Pre/Relation(Rel-Unr)	0.13	0.13	1.00	0.322
Post/Relation(Rel-Unr)	0.22	0.13	1.77	0.083
Pre/Interest	0.16	0.06	2.77	0.008**
Post/Interest	0.22	0.06	3.98	<.001***
Pre/Knowledge:Relation	-0.14	0.26	-0.57	0.574
Post/Knowledge:Relation	-0.20	0.26	-0.78	0.439
Pre/Knowledge:Interest	-0.01	0.07	-0.12	0.902
Post/Knowledge:Interest	0.05	0.07	0.77	0.441
Pre/Relation:Interest	-0.11	0.08	-1.47	0.147
Post/Relation:Interest	-0.16	0.08	-2.13	0.037*
Pre/Knowledg:Relation:Interest	0.11	0.14	0.78	0.434
Post/Knowledg:Relation:Interest	0.07	0.14	0.51	0.611
Random Effects	Variance	SD		
Participants	0.11	0.34		

Knowledge	-	-
Relation	0.06	0.24
Knowledge:Relation	-	-
Picture	0.46	0.68
Knowledge	-	-
Relation	0.18	0.42
Knowledge: Unrelated	0.28	0.53
Knowledge:Related	0.36	0.60
Residual	2.25	1.50

Notes. Pre = before knowledge acquisition, Post = after knowledge acquisition. Knowledge refers to the affective-social nature of the information, Neg-Neu = Negative-Neutral. Relation refers to the relatedness of information to picture content, Rel-Unr = Related-Unrelated. Interest = centered art interest covariate. "/" indicates nesting of fixed factors. ":" indicates interactions between fixed factors.

*** p <.001, ** p <.01, * p <.05.

Table S2.

Arousal rating: Effects of biographical knowledge, relatedness and covariate art interest before and after knowledge acquisition.

Variable	*b*	*SE*	*t*	*p*
Intercept	3.85	0.16	24.19	<.001***
Learning(Post-Pre)	-0.02	0.07	-0.26	0.796
Pre/Knowledge(Neg-Neu)	-0.06	0.11	-0.57	0.567
Post/Knowledge(Neg-Neu)	0.40	0.11	3.60	<.001***
Pre/Relation(Rel-Unr)	0.06	0.12	0.54	0.593
Post/Relation(Rel-Unr)	0.17	0.12	1.45	0.152
Pre/Interest	0.23	0.07	3.08	0.004**
Post/Interest	0.22	0.07	3.07	0.004**
Pre/Knowledge:Relation	-0.004	0.25	-0.02	0.988
Post/Knowledge:Relation	0.02	0.25	0.08	0.939
Pre/Knowledge:Interest	-0.05	0.07	-0.71	0.477
Post/Knowledge:Interest	-0.14	0.07	-1.94	0.053
Pre/Relation:Interest	-0.12	0.07	-1.67	0.095
Post/Relation:Interest	-0.12	0.07	-1.63	0.104
Pre/Knowledg:Relation:Interest	-0.12	0.14	-0.83	0.406
Post/Knowledg:Relation:Interest	-0.17	0.14	-1.21	0.225
Random Effects	Variance	SD		
Participants	0.24	0.49		
Knowledge	-	-		
Relation	-	-		

Knowledge:Relation	-	-
Picture	2.03	1.42
Knowledge	-	-
Relation	-	-
Neutral:Unrelated	1.44	1.20
Negative:Unrelated	1.54	1.24
Neutral:Related	1.98	1.41
Negative:Related	1.11	1.05
Residual	2.37	1.54

Notes. Pre = before knowledge acquisition, Post = after knowledge acquisition. Knowledge refers to the affective-social nature of the information, Neg-Neu = Negative-Neutral. Relation refers to the relatedness of information to picture content, Rel-Unr = Related-Unrelated. Interest= centered art interest covariate. "/" indicates nesting of fixed factors. ":" indicates interactions between fixed factors.

*** p <.001, ** p <.01.

Table S3.

Quality rating: Effects of biographical knowledge, relatedness and covariate art interest before and after knowledge acquisition.

Variable	*b*	*SE*	*t*	*p*
Intercept	4.59	0.18	25.64	<.001***
Learning(Post-Pre)	-0.18	0.06	-3.21	0.001**
Pre/Knowledge(Neg-Neu)	-0.04	0.08	-0.44	0.662
Post/Knowledge(Neg-Neu)	-0.13	0.08	-1.58	0.115
Pre/Relation(Rel-Unr)	0.02	0.14	0.17	0.864
Post/Relation(Rel-Unr)	0.01	0.14	0.10	0.920
Pre/Interest	0.07	0.09	0.77	0.445
Post/Interest	0.04	0.09	0.45	0.654
Pre/Knowledge:Relation	0.17	0.16	1.07	0.286
Post/Knowledge:Relation	0.01	0.16	0.07	0.942
Pre/Knowledge:Interest	0.07	0.06	1.10	0.272
Post/Knowledge:Interest	0.06	0.06	0.96	0.336
Pre/Relation:Interest	-0.11	0.06	-1.73	0.087
Post/Relation:Interest	-0.15	0.06	-2.36	0.020*
Pre/Knowledg:Relation:Interest	-0.15	0.12	-1.24	0.217
Post/Knowledg:Relation:Interest	-0.03	0.12	-0.26	0.798
Random Effects	Variance	Sd		
Participants	0.41	0.64		
Knowledge	-	-		
Relation	0.04	0.19		

Knowledge:Relation	-	-
Picture	0.59	0.77
Knowledge	-	-
Relation	0.35	0.59
Kowledge:Relation	-	-
Residual	1.66	1.29

Notes. Pre = before knowledge acquisition, Post = after knowledge acquisition. Knowledge refers to the affective-social nature of the information, Neg-Neu = Negative-Neutral. Relation refers to the relatedness of information to picture content, Rel-Unr = Related-Unrelated. Interest = centered art interest covariate. "/" indicates nesting of fixed factors. ":" indicates interactions between fixed factors.

*** p <.001, ** p <.01, * p <.05.

Table S4.

EPN amplitude (250-350ms): Effects of biographical knowledge, relatedness and covariate art interest.

Variable	*b*	*SE*	*t*	*p*
Intercept	-0.01	0.71	-0.02	0.986
Knowledge(Neg-Neu)	-0.27	0.11	-2.42	0.016*
Relation(Rel-Unr)	0.05	0.11	0.44	0.659
Interest	0.06	0.48	0.12	0.902
Knowledge:Relation	-0.02	0.22	-0.10	0.923
Knowledge:Interest	0.01	0.08	0.13	0.897
Relation:Interest	0.01	0.08	0.18	0.858
Knowledge:Relation:Interest	0.30	0.16	1.87	0.061
Random Effects	Variance	SD		
Participants	13.79	3.71		
Knowledge	-	-		
Relation	-	-		
Knowledge:Relation	-	-		
Picture	2.11	1.45		
Knowledge	-	-		
Relation	-	-		
Knowledge: Relation	-	-		
Residual	24.50	4.95		

Notes. Knowledge refers to the affective-social nature of the information, Neg-Neu = Negative-Neutral. Relation refers to the relatedness of information to picture content, Rel-

Unr = Related-Unrelated. Interest = centered art interest covariate. ":" indicates interactions between fixed factors. * $p < .05$.

Table S5.

LPP amplitude (400-700ms): Effects of biographical knowledge, relatedness and covariate art interest.

Variable	*b*	*SE*	*p*	Variable
Intercept	0.00	0.33	0.01	0.992
Knowledge(Neg-Neu)	0.13	0.10	1.25	0.213
Relation(Rel-Unr)	0.23	0.10	2.22	0.026*
Interest	0.02	0.22	0.10	0.918
Knowledge:Relation	-0.35	0.21	-1.67	0.094
Knowledge:Interest	0.19	0.08	2.37	0.018*
Relation:Interest	0.03	0.08	0.34	0.735
Knowledge:Relation:Interest	-0.10	0.15	-0.66	0.512
Random Effects	Variance	SD		
Participants	2.66	1.63		
Knowledge	-	-		
Relation	-	-		
Knowledge:Relation	-	-		
Picture	0.79	0.89		
Knowledge	-	-		
Relation	-	-		
Knowledge: Relation	-	-		
Residual	22.25	4.72		

Notes. Knowledge refers to the affective-social nature of the information, Neg-Neu = Negative-Neutral. Relation refers to the relatedness of information to picture content, Rel-

Unr = Related-Unrelated. Interest = centered art interest covariate. ":" indicates interactions between fixed factors. * $p < .05$.

Table S6.

Liking rating: effects of biographical knowledge and relatedness before and after knowledge acquisition

Variable	*b*	*SE*	*t*	*p*
Intercept	3.94	0.15	27.13	<.001***
Learning(Post-Pre)	-0.32	0.07	-4.84	<.001***
Pre/Knowledge(Neg-Neu)	-0.05	0.11	-0.46	0.646
Post/Knowledge(Neg-Neu)	-0.36	0.11	-3.41	0.001**
Pre/Relation(Rel-Unr)	0.13	0.13	0.97	0.336
Post/Relation(Rel-Unr)	0.22	0.13	1.72	0.091
Pre/(Knowledge:Relation)	-0.14	0.26	-0.57	0.575
Post/(Knowledge:Relation)	-0.20	0.26	-0.78	0.440
Random Effects	Variance	*SD*		
Participants	0.18	0.42		
Knowledge	-	-		
Relation	0.09	0.29		
Knowledge:Relation	-	-		
Picture	0.46	0.68		
Knowledge	0.08	0.28		
Relation	0.18	0.42		
Knowledge:Relation	0.97	0.99		
Residual	2.25	1.50		

Notes. Pre = before knowledge acquisition, Post = after knowledge acquisition. Knowledge refers to the affective-social nature of the information, Neg-Neu = Negative-Neutral. Relation

refers to the relatedness of information to picture content, Rel-Unr = Related-Unrelated. "/" indicates nesting of fixed factors. ":" indicates interactions between fixed factors.

*** p <.001, ** p <.01.

Table S7.

Arousal rating: effects of biographical knowledge and relatedness before and after knowledge acquisition.

Variable	*b*	*SE*	*t*	*p*
Intercept	3.85	0.17	23.02	<.001***
Learning(Post-Pre)	-0.02	0.07	-0.26	0.796
Pre/Knowledge(Neg-Neu)	-0.06	0.11	-0.57	0.567
Post/Knowledge(Neg-Neu)	0.40	0.11	3.60	<.001***
Pre/Relation(Rel-Unr)	0.06	0.13	0.51	0.610
Post/Relation(Rel-Unr)	0.17	0.13	1.39	0.172
Pre/(Knowledge:Relation)	-0.004	0.25	-0.02	0.988
Post/(Knowledge:Relation)	0.02	0.25	0.08	0.939
**Random Effects**	**Variance**	***SD***		
Participants	0.33	0.57		
Knowledge	-	-		
Relation	0.05	0.23		
Knowledge:Relation	-	-		
Picture	0.53	0.73		
Knowledge	0.11	0.33		
Relation	0.16	0.40		
Knowledge:Relation	0.88	0.94		
Residual	2.37	1.54		

Notes. Pre = before knowledge acquisition, Post = after knowledge acquisition. Knowledge refers to the affective-social nature of the information, Neg-Neu = Negative-Neutral. Relation

refers to the relatedness of information to picture content, Rel-Unr = Related-Unrelated. "/" indicates nesting of fixed factors. ":" indicates interactions between fixed factors.

*** $p <.001$.

Table S8.

Quality judgment: effects of biographical knowledge and relatedness before and after knowledge acquisition.

Variable	*b*	*SE*	*t*	*p*
Intercept	4.59	0.18	25.83	<.001***
Learning(Post-Pre)	-0.18	0.06	-3.23	0.001**
Pre/Knowledge(Neg-Neu)	-0.04	0.08	-0.42	0.678
Post/Knowledge(Neg-Neu)	-0.13	0.08	-1.50	0.134
Pre/Relation(Rel-Unr)	0.02	0.14	0.17	0.867
Post/Relation(Rel-Unr)	0.01	0.14	0.10	0.922
Pre/(Knowledge:Relation)	0.17	0.18	0.98	0.330
Post/(Knowledge:Relation)	0.01	0.18	0.07	0.947
Random Effects	Variance	*SD*		
Participants	0.40	0.63		
Knowledge	-	-		
Relation	0.07	0.26		
Knowledge:Relation	-	-		
Picture	0.59	0.77		
Knowledge	-	-		
Relation	0.35	0.60		
Knowledge: Unrelated	0.11	0.33		
Knowledge: Related	0.02	0.14		
Residual	1.64	1.28		

Notes. Pre = before knowledge acquisition, Post = after knowledge acquisition. Knowledge refers to the affective-social nature of the information, Neg-Neu = Negative-Neutral. Relation refers to the relatedness of information to picture content, Rel-Unr = Related-Unrelated. "/" indicates nesting of fixed factors. ":" indicates interactions between fixed factors.

*** p <.001, ** p <.01.

Table S9.

Liking rating: biographical knowledge nested in time of rating (pre vs. post knowledge acquisition) nested in relatedness factor.

Variable	*b*	*SE*	*t*	*p*
Intercept	3.94	0.14	27.17	<.001***
Relation(Rel-Unr)	0.18	0.07	2.58	0.010**
Unr/Learning(Post-Pre)	-0.37	0.10	-3.83	<.001***
Rel/Learning(Post-Pre)	-0.27	0.10	-2.82	0.005**
Unr/Pre/Knowledge(Neg-Neu)	0.02	0.14	0.16	0.871
Rel/Pre/Knowledge(Neg-Neu)	-0.12	0.14	-0.84	0.401
Unr/Post/Knowledge(Neg-Neu)	-0.26	0.14	-1.82	0.070
Rel/Post/Knowledge(Neg-Neu)	-0.46	0.14	-3.20	0.002**
Random Effects	Variance	SD		
Participants	0.18	0.42		
Knowledge	-	-		
Picture	0.46	0.68		
Knowledge	0.07	0.26		
Residual	2.37	1.54		

Notes. Pre = before knowledge acquisition, Post = after knowledge acquisition. Knowledge refers to the affective-social nature of the information, Neg-Neu = Negative-Neutral. Relation refers to the relatedness of information to picture content, Rel-Unr = Related-Unrelated. "/" indicates nesting of fixed factors.

*** *p* <.001, ** p <.01.

Table S10.

Arousal rating: biographical knowledge nested in time of rating (pre vs. post knowledge acquisition) nested in relatedness factor.

Variable	*b*	*SE*	*t*	*p*
Intercept	3.85	0.17	23.09	<.001***
Relation(Rel-Unr)	0.12	0.07	1.72	0.086
Unr/Learning(Post-Pre)	-0.07	0.10	-0.74	0.462
Rel/Learning(Post-Pre)	0.04	0.10	0.38	0.705
Unr/Pre/Knowledge(Neg-Neu)	-0.06	0.15	-0.41	0.681
Rel/Pre/Knowledge(Neg-Neu)	-0.07	0.15	-0.44	0.663
Unr/Post/Knowledge(Neg-Neu)	0.39	0.15	2.60	0.010*
Rel/Post/Knowledge(Neg-Neu)	0.41	0.15	2.73	0.007**
Random Effects	Variance	SD		
Participants	0.32	0.57		
Knowledge	0.02	0.13		
Picture	0.53	0.73		
Knowledge	0.10	0.32		
Residual	2.47	1.57		

Notes. Pre = before knowledge acquisition, Post = after knowledge acquisition. Knowledge refers to the affective-social nature of the information, Neg-Neu = Negative-Neutral. Relation refers to the relatedness of information to picture content, Rel-Unr = Related-Unrelated. "/" indicates nesting of fixed factors.

*** *p* <.001, ** p <.01, * p <.05.

Table S11.

Quality judgment: biographical knowledge nested in time of rating (pre vs. post knowledge acquisition) nested in relatedness factor.

Variable	*b*	*SE*	*t*	*p*
Intercept	4.59	0.18	25.57	<.001***
Relation(Rel-Unr)	0.02	0.06	0.32	0.751
Unr/Learning(Post-Pre)	-0.18	0.08	-2.15	0.032*
Rel/Learning(Post-Pre)	-0.19	0.08	-2.27	0.024*
Unr/Pre/Knowledge(Neg-Neu)	-0.12	0.12	-1.02	0.306
Rel/Pre/Knowledge(Neg-Neu)	0.05	0.12	0.43	0.668
Unr/Post/Knowledge(Neg-Neu)	-0.13	0.12	-1.12	0.262
Rel/Post/Knowledge(Neg-Neu)	-0.12	0.12	-1.03	0.306
Random Effects	Variance	SD		
Participants	0.42	0.65		
Knowledge	-	-		
Picture	0.59	0.77		
Knowledge	0.01	0.09		
Residual	1.75	1.32		

Notes. Pre = before knowledge acquisition, Post = after knowledge acquisition. Knowledge refers to the affective-social nature of the information, Neg-Neu = Negative-Neutral. Relation refers to the relatedness of information to picture content, Rel-Unr = Related-Unrelated. "/" indicates nesting of fixed factors.

*** *p* <.001, * p <.05.

Table S12.

Liking rating: Effects of biographical knowledge, relatedness and trial repetitions.

Variable	*b*	*SE*	*t*	*p*
Intercept	3.72	0.16	22.70	<.001***
Knowledge(Neg-Neu)	-0.37	0.11	-3.51	<.001***
Relation(Rel-Unr)	0.22	0.16	1.33	0.188
Rep	-0.01	0.01	-1.20	0.231
Knowledge:Relation	-0.08	0.26	-0.29	0.773
Knowledge:Rep	0.01	0.01	0.37	0.708
Relation:Rep	0.01	0.01	0.49	0.628
Knowledge:Relation:Rep	0.0007	0.03	0.03	0.980
Random Effects	Variance	*SD*		
Participants	0.36	0.60		
Knowledge	0.15	0.38		
Relation	0.32	0.57		
Knowledge:Relation	0.59	0.77		
Picture	0.50	0.71		
Knowledge	-	-		
Relation	0.49	0.70		
Knowledge:Unrelated	0.52	0.72		
Knowledge:Related	0.60	0.77		
Residual	2.07	1.44		

Notes. Knowledge refers to the affective-social nature of the information, Neg-Neu = Negative-Neutral. Relation refers to the relatedness of information to picture content, Rel-Unr = Related-Unrelated. Rep refers to the number of trial repetitions ":" indicates interactions between fixed factors. *** p <.001.

Table S13.

Effects of biographical knowledge and relatedness on EPN amplitude (250-350ms).

Variable	*b*	*SE*	*t*	*p*
Intercept	-0.01	0.70	-0.02	0.985
Knowledge(Neg-Neu)	-0.27	0.11	-2.32	0.022*
Relation(Rel-Unr)	0.05	0.13	0.36	0.720
Knowledge:Relation	-0.02	0.29	-0.08	0.936
Random Effects	Variance	*SD*		
Participants	13.33	3.65		
Knowledge	-	-		
Relation	-	-		
Knowledge:Relation	-	-		
Picture	0.00005	0.01		
Knowledge	-	-		
Relation	-	-		
Neutral:Unrelated	1.86	1.36		
Negative:Unrelated	2.45	1.57		
Neutral:Related	2.71	1.65		
Negative:Related	2.02	1.42		
Residual	24.38	4.94		

Notes. Knowledge refers to the affective-social nature of the information, Neg-Neu = Negative-Neutral. Relation refers to the relatedness of information to picture content, Rel-Unr = Related-Unrelated. ":" indicates interactions between fixed factors. * p <.05.

Table S14.

Effects of biographical knowledge and relatedness on LPP amplitude (400-700ms).

Variable	*b*	*SE*	*t*	*p*
Intercept	0.003	0.33	0.01	0.993
Knowledge(Neg-Neu)	0.13	0.13	1.05	0.303
Relation(Rel-Unr)	0.23	0.14	1.64	0.110
Knowledge:Relation	-0.35	0.25	-1.38	0.174
Random Effects	Variance	*SD*		
Participants	2.57	1.60		
Knowledge	0.12	0.34		
Relation	-	-		
Knowledge:Relation	-	-		
Picture	0.21	0.45		
Knowledge	-	-		
Relation	-	-		
Neutral:Unrelated	1.79	1.34		
Negative:Unrelated	1.96	1.40		
Neutral:Related	1.33	1.15		
Negative:Related	1.69	1.30		
Residual	22.11	4.70		

Notes. Knowledge refers to the affective-social nature of the information, Neg-Neu = Negative-Neutral. Relation refers to the relatedness of information to picture content, Rel-Unr = Related-Unrelated. ":" indicates interactions between fixed factors.

Table S15.

Effects of biographical knowledge, relatedness and reaction time on EPN amplitude (250-350ms).

Variable	*b*	*SE*	*t*	*p*
Intercept	-0.01	0.70	-0.02	0.987
Knowledge(Neg-Neu)	-0.27	0.12	-2.31	0.023*
Relation(Rel-Unr)	0.05	0.13	0.34	0.735
RT	0.00	0.00	2.27	0.023*
Knowledge:Relation	-0.02	0.29	-0.06	0.953
Knowledge:RT	0.00	0.00	0.65	0.518
Relation:RT	0.00	0.00	0.48	0.632
Knowledge:Relation:RT	0.00	0.00	1.62	0.106
Random Effects	Variance	*SD*		
Participants	13.49	3.67		
Knowledge	-	-		
Relation	-	-		
Knowledge:Relation	-	-		
Picture	1.55	1.25		
Knowledge	-	-		
Relation	-	-		
Neutral:Unrelated	3.57	1.89		

Negative:Unrelated	2.92	1.71
Neutral:Related	3.50	1.87
Negative:Related	3.82	1.96
Residual	24.37	4.94

Notes. Knowledge refers to the affective-social nature of the information, Neg-Neu = Negative-Neutral. Relation refers to the relatedness of information to picture content, Rel-Unr = Related-Unrelated. RT refers to reaction time (ms) ":" indicates interactions between fixed factors. * p <.05.

Table S16.

Effects of biographical knowledge, relatedness and trial repetitions on EPN amplitude (250-350ms).

Variable	*b*	*SE*	*t*	*p*
Intercept	-0.01	0.70	-0.02	0.985
Knowledge(Neg-Neu)	-0.27	0.11	-2.42	0.016*
Relation(Rel-Unr)	0.05	0.11	0.44	0.662
Rep	-0.03	0.02	-1.14	0.255
Knowledge:Relation	-0.02	0.22	-0.10	0.921
Knowledge:Rep	0.04	0.05	0.80	0.422
Relation:Rep	-0.04	0.05	-0.87	0.384
Knowledge:Relation:Rep	0.08	0.10	0.85	0.397
Random Effects	Variance	*SD*		
Participants	13.35	3.65		
Knowledge	-	-		
Relation	-	-		
Knowledge:Relation	-	-		
Picture	2.12	1.46		
Knowledge	-	-		
Relation	-	-		
Knowledge:Relation	-	-		

Residual	24.50	4.95

Notes. Knowledge refers to the affective-social nature of the information, Neg-Neu = Negative-Neutral. Relation refers to the relatedness of information to picture content, Rel-Unr = Related-Unrelated. Rep refers to number of trial repetitions":" indicates interactions between fixed factors. * p <.05.

Table S17.

Effects of biographical knowledge, relatedness and reaction time on LPP amplitude (400-700ms).

Variable	*b*	*SE*	*t*	*p*
Intercept	0.002	0.33	0.01	0.996
Knowledge(Neg-Neu)	0.13	0.13	0.98	0.334
Relation(Rel-Unr)	0.24	0.14	1.69	0.100
RT	-0.0002	0.00007	-2.82	0.005**
Knowledge:Relation	-0.35	0.25	-1.38	0.174
Knowledge:RT	0.0001	0.0001	-1.03	0.303
Relation:RT	0.000005	0.0001	0.04	0.972
Knowledge:Relation:RT	0.00001	0.0003	0.05	0.960
Random Effects	Variance	*SD*		
Participants	2.60	1.61		
Knowledge	0.14	0.37		
Relation	-	-		
Knowledge:Relation	-	-		
Picture	0.35	0.59		
Knowledge	-	-		
Relation	-	-		
Neutral:Unrelated	1.85	1.36		

Negative:Unrelated	2.07	1.44
Neutral:Related	1.51	1.23
Negative:Related	1.82	1.35
Residual	22.09	4.70

Notes. Knowledge refers to the affective-social nature of the information, Neg-Neu = Negative-Neutral. Relation refers to the relatedness of information to picture content, Rel-Unr = Related-Unrelated. RT refers to reaction time (ms) ":" indicates interactions between fixed factors. ** p <.01.

Table S18

Effects of biographical knowledge, relatedness and trial repetitions on LPP amplitude (400-700ms).

Variable	*b*	*SE*	*t*	*p*
Intercept	0.003	0.33	0.01	0.992
Knowledge(Neg-Neu)	0.13	0.13	1.05	0.300
Relation(Rel-Unr)	0.23	0.14	1.64	0.110
Rep	0.06	0.02	2.49	0.013*
Knowledge:Relation	-0.35	0.25	-1.38	0.174
Knowledge:Rep	0.07	0.05	1.52	0.128
Relation:Rep	0.03	0.05	0.68	0.498
Knowledge:Relation:Rep	-0.04	0.09	-0.45	0.650
Random Effects	Variance	*SD*		
Participants	2.58	1.60		
Knowledge	0.12	0.34		
Relation	-	-		
Knowledge:Relation	-	-		
Picture	0.12	0.35		
Knowledge	-	-		
Relation	-	-		
Neutral:Unrelated	1.54	1.24		

Negative:Unrelated	1.77	1.33
Neutral:Related	1.27	1.13
Negative:Related	1.53	1.24
Residual	22.10	4.70

Notes. Knowledge refers to the affective-social nature of the information, Neg-Neu = Negative-Neutral. Relation refers to the relatedness of information to picture content, Rel-Unr = Related-Unrelated. Rep refers to number of trial repetitions":" indicates interactions between fixed factors. * p <.05.

Table S19.

EPN amplitude (250-350ms): biographical knowledge nested in relatedness factor.

Variable	*b*	*SE*	*t*	*p*
Intercept	-0.01	0.70	-0.02	0.985
Relation(Rel-Unr)	0.05	0.11	0.44	0.661
Unr/Knowledge(Neg-Neu)	-0.25	0.16	-1.62	0.107
Rel/Knowledge(Neg-Neu)	-0.28	0.16	-1.76	0.081
Random Effects	Variance	*SD*		
Participants	13.34	3.65		
Knowledge	0.02	0.12		
Picture	2.12	1.45		
Knowledge	-	-		
Residual	24.49	4.95		

Notes. Knowledge refers to the affective-social nature of the information, Neg-Neu = Negative-Neutral. Relation refers to the relatedness of information to picture content, Rel-Unr = Related-Unrelated. "/" indicates nesting of fixed factors.

Table S20.

LPP amplitude (400-700ms): biographical knowledge nested in relatedness factor.

Variable	*b*	*SE*	*t*	*p*
Intercept	0.003	0.33	0.01	0.992
Relation(Rel-Unr)	0.23	0.10	2.22	0.026*
Unr/Knowledge(Neg-Neu)	0.31	0.16	1.92	0.058
Rel/Knowledge(Neg-Neu)	-0.04	0.16	-0.28	0.781
Random Effects	Variance	*SD*		
Participants	2.58	1.61		
Knowledge	0.11	0.33		
Picture	0.79	0.89		
Knowledge	-	-		
Residual	22.23	4.72		

Notes. Knowledge refers to the affective-social nature of the information, Neg-Neu = Negative-Neutral. Relation refers to the relatedness of information to picture content, Rel-Unr = Related-Unrelated. "/" indicates nesting of fixed factors. * p <.05.
